# Running and Thinking: Unmasking the Lingering Effects of Sports Concussions Through Complex Dual-Task Testing

**DOI:** 10.3390/sports13050144

**Published:** 2025-05-12

**Authors:** Gabriel Lavoie, Mathieu Bolduc, Veronik Sicard, Franco Lepore, Dave Ellemberg

**Affiliations:** 1 Department of Psychology, Université de Montréal, Montreal, QC H3T 1J4, Canada; lavoiegabriel1234@hotmail.com (G.L.); mathieu.bolduc.2@umontreal.ca (M.B.); franco.lepore@umontreal.ca (F.L.); 2 Children’s Hospital of Eastern Ontario Research Institute, Ottawa, ON K1H 5B2, Canada; vsicard@cheo.on.ca; 3 School of Kinesiology and Physical Activity Sciences, Université de Montréal, Montreal, QC H3T 1J4, Canada

**Keywords:** mild traumatic brain injury, cognition, gait

## Abstract

Objective: This study investigated gait and cognitive dual-task costs under a dual-task paradigm that was more challenging than the traditional tasks used in research. Methods: A total of 43 18–25-year-old male and female student athletes were recruited (20 asymptomatic concussed athletes who suffered at least one concussion 2.79–7.92 months before testing, 23 never concussed). Athletes performed a complex rapid decision-making and executive function computerized task while walking continuously and maintaining a predetermined speed on a non-motorized treadmill (6.5 km/h). The outcome measures were gait and cognitive dual-task costs. Results: Repeated-measures ANOVAs were conducted to evaluate group differences. Pearson correlations were conducted to evaluate the association between dual-task costs and concussion injury variables. The results showed that both groups experienced greater difficulty with dual-task performance related to gait, whereas only the concussion group exhibited poorer cognitive performance under the dual-task condition (both not significant). The significant correlation between time since injury and gait dual-task cost (r = −0.72, *p* < 0.001) indicated that athletes with a more recent concussion increased their gait speed whilst athletes with an older concussion reduced their gait speed during the dual-task. Moreover, the cognitive dual-task cost was significantly correlated to symptom recovery (r = 0.461, *p* = 0.047), suggesting that a longer recovery time from concussion is associated with an increased dual-task cost. Conclusions: While dual-task gait alterations were common to both groups, only individuals with a history of concussion showed specific cognitive impairments under dual-task conditions. The observed associations between dual-task costs and both time since injury and symptom recovery underscore the potential of complex dual-task assessments to provide nuanced insights into post-concussion recovery trajectories and to detect subtle, lingering deficits.

## 1. Introduction

Sport-related concussions can have significant consequences for an athlete’s personal, academic, and professional lives. These may worsen if the return to play or to intellectual activities is premature and not properly planned [1]. This has been the driving force behind much of the research aimed at identifying sensitive assessment measures to guide the decision-making process regarding the timing of return to play and return to school or work. Sports concussions can lead to physical, psychological, and cognitive symptoms [1]. Cognitive functioning in the acute and chronic phases of an injury has received the most attention, with studies suggesting that learning, memory, attention, and executive functions are affected in the days following a concussion [2]. Executive functions appear to have the most protracted recovery in otherwise asymptomatic athletes [3]. Accumulating evidence suggests that cognitive deficits persist after symptom resolution [4]. The days since last concussion or even the number of concussions could impact cognitive recovery [5]. Given the low reliability of symptom reports, the assessment of cognitive functioning has gained in popularity as a means of tracking recovery following a sports concussion and managing a safe return to intellectual (i.e., return to learn/work) and physical activities [1]. Combining cognitive testing with symptom assessment provides greater sensitivity than assessing symptoms alone [1].

Over the past two decades, the cognitive evaluation of athletes during the acute phase of injury has become increasingly common, prompting the creation of several commercial computerized test batteries (e.g., ImPACT, ANAM, Cogstate, CogSport). These computerized tools are considered by some to offer multiple benefits over traditional paper-and-pencil methods, such as the ability to administer tests repeatedly, simplified administration, and reduced time requirements for the test administrator [6]. They are usually sensitive enough to detect differences in the days following the concussion, while athletes are still symptomatic, but they lose this sensitivity in asymptomatic athletes. This may have reinforced the notion that cognitive deficits subside within the same 14- to 21-day period as symptoms following a concussion [7]. However, the empirical data do not fully support relying solely on computerized test batteries to determine safe return-to-play decisions, as these tools demonstrate limited validity and reliability as well as appear insensitive to the lingering effects of concussion once symptoms have resolved [4]. The complexity and level of difficulty of the tests might not always be suited for highly educated student athletes, which could sometimes lead to a ceiling effect [8].

An increasing number of studies indicate that long-term changes, particularly in executive function, can persist for months or even years following a concussion [2,3]. For example, several studies report persisting deficits using a switch task in asymptomatic athletes with a history of concussion [9,10]. For example, previous work found a significant reduction in performance accuracy on a complex visual switch task in a subgroup of asymptomatic university athletes who were tested approximately 2 years after their last concussion [10].

Sports often require simultaneous cognitive and motor demands, such as strategy planning while running. Dividing attention between these processes may heighten the risk of symptom recurrence or injury. Reviews have shown that dual-task gait assessments reveal lingering deficits in athletes after concussion [11,12], likely due to competition for limited cognitive resources. While useful, traditional dual-task paradigms have limitations, often relying on short, self-paced walking tasks (6–15 m) [11], which do not reflect the continuous, high-speed movement seen in sports like hockey or soccer. For example, in a study using an 18 m self-paced walking task combined with a simple arithmetic task, only the concussed group (only five participants) showed increased dual-task gait cost solely when the task was more complex [13].

Furthermore, the cognitive tasks employed in dual-task paradigms previously published may not adequately capture the complexity of the decision making that athletes face during a competitive sport. For example, a study evaluating 14 athletes with concussion found altered self-paced speed over a few meters when simultaneously performing a question-and-answer task (counting backwards from a random number, reciting the months of the year backwards, and spelling five-letter words backwards) [14]. Efficient decision making during competitive sports is more likely to require high-level executive functions [15]. Some studies have used more complex cognitive tasks, like the Stroop, which solicits cognitive inhibition [16,17]. For example, adolescents tested on average two months after a concussion performed more poorly on a self-paced walking task and the auditory Stroop, and they had a greater dual-task cost when compared to a control group [16]. These results suggest that more challenging cognitive tasks employed during dual-task paradigms may better identify altered cognitive functioning after concussion and not just gait alteration. The co-occurrence of a demanding continuous motor task and a complex cognitive task has yet to be used to study dual-task performance in athletes with a history of concussion.

Accordingly, the objective of this study was to compare the gait and cognitive outcomes of athletes with concussion with control athletes under single-task and dual-task conditions. It was hypothesized that individuals with concussion would exhibit greater dual-task costs compared to controls. The gait performance cost would be greater since previous studies showed that, under limited attentional resources, more complex tasks are prioritized over easier ones [13,14,18]. The secondary objective was to explore the relationship between the concussion-related variables (i.e., months since injury, symptoms intensity and number of symptoms at time of injury, days to symptoms recovery) and dual-task costs. It was hypothesized that concussion-related variables (i.e., time since injury, time to symptoms recovery, symptoms intensity) would be correlated to gait and cognitive dual-task costs.

## 2. Materials and Methods

### 2.1. Participants

To recruit participants, athletic directors and team coaches from seven colleges and three universities in the Montréal area were contacted. Additionally, participants helped recruit by referring their teammates to the study. A total of 43 18–25-year-old male and female student athletes were recruited (20 asymptomatic concussed athletes who suffered at least one concussion 2.79–7.92 months before testing, 23 never concussed). Four additional participants withdrew from this study. Two withdrew without giving further notice, one moved several km away unexpectedly, and another sustained a sports-related injury that prevented them from completing our task.

Individuals were excluded from participation if they had any known psychiatric, neurological, or neurodevelopmental disorders; a history of head trauma unrelated to sports concussions (or any head trauma in the case of control participants); current use of psychoactive medications; color blindness; a history of illicit substance use (e.g., stimulants, opiates, marijuana, sedatives); or reported consuming more than ten alcoholic drinks per week.

Three self-report questionnaires (Beck Anxiety Inventory [BAI]; Beck Depression Inventory-II [BDI-II]; Wender-Utah Rating Scale [WURS]) were used to screen participants that could score within clinical ranges of anxiety (BAI score ≥ 17), depression (BDI-II score ≥ 20), and ADHD (WURS score ≥ 47).

All athletes were asked: “Following a blow to the head, neck or body, have you ever experienced any concussion-like symptoms?” They were then presented with the list of clinical symptoms from the Sports Concussion Assessment Tool (SCAT-5). Anyone from the control group who responded “yes” was excluded, reducing the likelihood that an athlete with an undocumented concussion was placed in the control group.

Only athletes with at least one medically diagnosed sports-related concussion were included in the concussion group. At the time of testing, all participants were asymptomatic and had fully returned to both sports and school activities.

### 2.2. Procedures

This study was approved by the clinical research ethics committee of the Université of Montréal and followed the 1964 Declaration of Helsinki (CERC-21-008-D). All participants provided written informed consent.

This study involved two sessions, each on a separate day. Written informed consent was obtained at the beginning of each first session. One session consisted of the cognitive task only (cognitive single-task session) and the other consisted of the gait single-task alone as well as the combined gait and cognitive tasks (dual-task session). Each participant completed all conditions scheduled across the two testing sessions. To reduce any potential learning effect or impact of cognitive fatigue, the sessions were counterbalanced across both groups. Thus, half of the participants took part in the cognitive single-task session first, whilst the other half took part in the dual-task session first. Testing sessions were scheduled based on participant availability; participants were instructed to choose a time at which they felt well rested and had sufficient energy to complete the tasks adequately.

At the beginning of the first session, a structured interview and screening questionnaires were completed to ensure that all participants respected the inclusion criteria and to gather demographic and injury characteristics.

#### 2.2.1. Cognitive Single-Task Session

This session started by asking participants to complete the switch task while standing on a treadmill but without moving. During the task, participants were instructed to answer as quickly and as precisely as possible. Including practices and transitions, the cognitive task was completed in approximately 17 min.

#### 2.2.2. Dual-Task Session

Gait single task. Participants were first asked to accelerate and decelerate their walking speed to familiarize themselves with the non-motorized treadmill. When they became comfortable walking on the treadmill, they had to maintain a walking speed of 6.5 km/h (displayed on the indicator panel). The gait single-task lasted 3 min.

Dual tasks. After completing the gait single-task, participants completed the dual tasks, i.e., they simultaneously executed the gait task and the switch task (cognitive task). The dual tasks lasted 17 min (2 homogenous blocks of 2 min 10 s 2 heterogenous blocks of 4 min 20 s and some practices). All participants completed these two tasks in the same order because the gait single-task also served as a way of familiarization with the treadmill.

### 2.3. Questionnaires and Instruments

#### 2.3.1. Questionnaires

A structured interview was used to gather demographic data, such as age, sex, education, and sports, as well as concussion injury characteristics (date of injury, symptom duration, etc.). All participants with a history of concussion completed the SCAT-5 Post-Concussion Symptom Scale retrospectively to determine symptom severity at the time of injury. The following screening questionnaires were administered in no particular order:

The Beck Anxiety Inventory (BAI) is a 21-item self-report questionnaire that measures the severity of common anxiety symptoms over the past week (range = 0–63), with higher scores indicating more severe anxiety symptoms.

The Beck Depression Inventory (BDI) is a 21-item self-report questionnaire that measures the severity of depression symptoms over the past two weeks (range = 0–63), with higher scores indicating more severe depression symptoms.

The Wender–Utah Rating Scale (WURS) is a 25-item self-report questionnaire that retrospectively assesses the presence of ADHD symptoms during childhood (range = 0–100), with higher scores indicating more ADHD symptoms.

#### 2.3.2. Curve Trainer Woodway Treadmill

A non-motorized treadmill was utilized (Curve Trainer Woodway Treadmill, Woodway, Waukesha, WI, USA-manufactured) as it allowed participants to control their speed, making it easier to measure gait speed as an outcome. Woodway CurvePro 1.5 software provided 75 speed data points each second, allowing a precise measure of gait speed.

#### 2.3.3. Switch Task—Color/Shape Version

**Figure 1:**
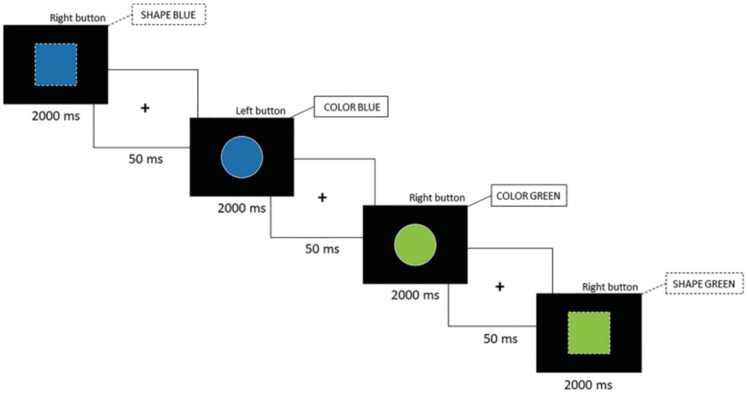
Color/shape switch task. This figure depicts the color/shape switch task used in the current study. The call-out states the ruleset used to elicit the appropriate response. For example, the first stimulus shows a blue square with a dashed outline. Therefore, the participants had to respond according to the shape, hence the right button, as already published in our previous work [21].

The color/shape switch task used in this study is based on a task developed and utilized in previous studies conducted by our laboratory [19,20]. The switch task consisted of three distinct conditions, each involving a different set of rules. The first two conditions were homogeneous conditions (60 trials/condition), during which participants had to respond either according to the color (green or blue) or according to the shape (square or circle). In the heterogeneous condition (120 trials), the participants’ responses were cued by the stimulus outline and were able to alternate quickly between them (solid = color rule; dashed = shape rule). See Figure 1 for a visual representation of the task. An alternative version was used to reduce practice effects, reversing rulesets to minimize repeated testing practice effects [19].

### 2.4. Outcome Measures

#### 2.4.1. Gait Outcomes

Gait speed was measured as the average speed (km/h) maintained during both the gait single-task and dual-task walking conditions. The gait dual-task cost was calculated by taking the absolute difference between the single-task and the dual-task gait speeds. A higher gait dual-task cost indicates a decrease in gait speed stability when a secondary task is added, reflecting the task’s impact on walking efficiency.

To further quantify this effect, the relative impact of gait dual-task cost was computed. This measure, expressed as a percentage, illustrates the proportional decrease in gait speed during the dual tasks compared to the single task.

relative impact of dual tasks on gait = ((Gait dual-task cost)/(Single-task gait speed)) × 100 (1)

Expressing this measure in percentages allows for a direct comparison with cognitive outcomes, facilitating an integrated analysis of motor and cognitive dual-task interferences.

#### 2.4.2. Cognitive Outcomes

The inverse efficiency score (IES) was calculated by dividing reaction time and accuracy in the heterogeneous condition of the switch task. The dual-task cognitive cost was calculated by subtracting the single-task IES from the dual-task IES. A positive value indicates a decline in cognitive performance under the dual-task condition (reflecting increased cognitive load), whereas a negative value suggests improved performance under the dual-task condition.

The relative impact of the cognitive dual tasks was calculated and is expressed in percentages, mirroring the method used for the gait outcomes.

relative impact of dual tasks on cognition = ((Cognitive dual-task cost)/(Single-task IES)) × 100(2)

### 2.5. Statistical Analyses

Demographic (age, sex, years of education, current level of education, height, sports) and injury-related (months since injury, symptom intensity and number of symptoms at time of injury, days to symptom resolution, number of previous diagnosed and undiagnosed concussions) information was summarized with descriptive statistics. Demographic information was compared between groups using independent sample t-tests or chi-square tests, as appropriate. Moreover, independent sample t-tests were used to compare BDI, BAI, and WURS between groups.

Gait speed and cognitive IES were analyzed with 2 × 2 (groupxession) repeated-measures ANOVAs. Post hoc analyses were conducted even in the absence of an interaction as an exploratory analysis. Gait and cognitive dual-task costs were compared between groups with independent sample t-tests. Pearson correlations were conducted within the concussion group to explore the association between gait and cognitive dual-task costs and injury-related characteristics.

A significance level of 0.05 (α ˂ 0.05) was employed for all statistical analyses. Analyses were conducted using SPSS 28.0 (IBM, Armonk, NY, USA).

## 3. Results

### 3.1. Participant Demographics

Demographic and injury-related information is presented in Table 1. Forty-three college and university athletes participated in this study. Of those, 20 athletes had at least one medically diagnosed concussion (concussion group: 2.79–7.92 months prior to testing, [mean ± SD = 5.25 ± 0.32 months]), and 23 were never diagnosed with a concussion nor did they self-report concussion (control group). A significant between-group difference was found for years of education (*p* = 0.032). However, this variable was not correlated to any of our outcome measures (ps ≥ 0.162), thus should not have significantly impacted our main findings. No other group differences were found in any other demographic characteristic (ps ≥ 0.232). Except for two athletes who took 4 months to recover, athletes with a concussion achieved a full recovery within the first 3 weeks after injury.

### 3.2. Gait Outcomes

**Figure 2:**
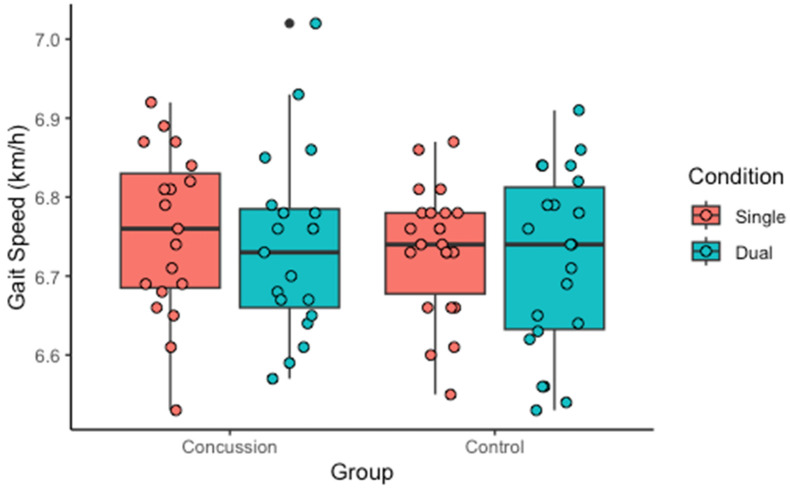
Average gait speed during the single-task and dual-task conditions for the concussion and control groups.

No groupxcondition interaction was found for gait speed, *p* = 0.899. Post hoc analyses showed no effect of the condition within the concussion (*p* = 0.457) or control groups (*p* = 0.650) or between-group differences under both conditions (single task: *p* = 0.514; dual tasks: *p* = 0.599), as shown in Figure 2.

No significant difference was found in the gait dual-task cost between the concussion and control groups (*p* = 0.32). The relative impact of the dual tasks on gait was 1.19% for the concussion group and 1.49% for the control group.

**Figure 3:**
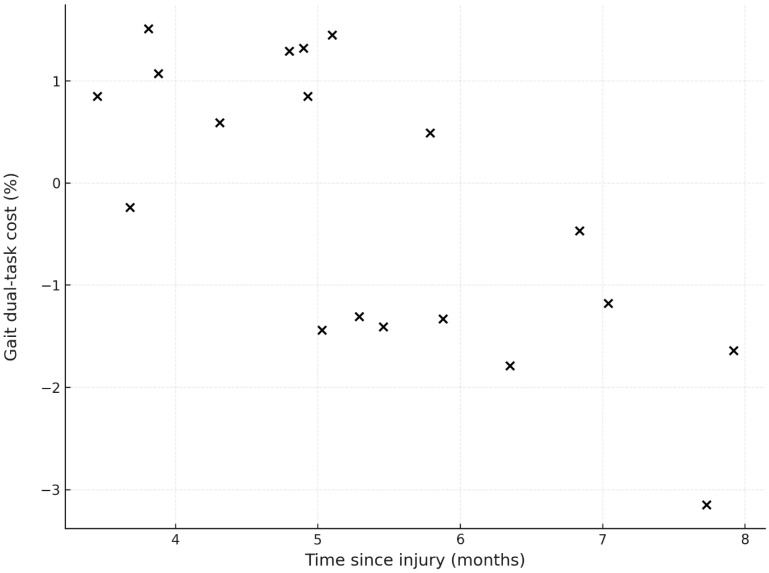
Relationship between months since concussion and gait dual-task cost. A positive gait dual-task cost indicates an increase in gait speed under the dual-task condition, whereas a negative cost reflects a reduction.

Within the concussion group, a strong association was found between gait dual-task cost and months since injury when the change in direction of the speed was taken into consideration (r = −0.72, *p* < 0.001; Figure 3), with more recent concussions being associated with increased speed. No other associations were found between gait dual-task costs and injury-related characteristics (ps ≥ 0.250).

### 3.3. Cognitive Outcomes

**Figure 4:**
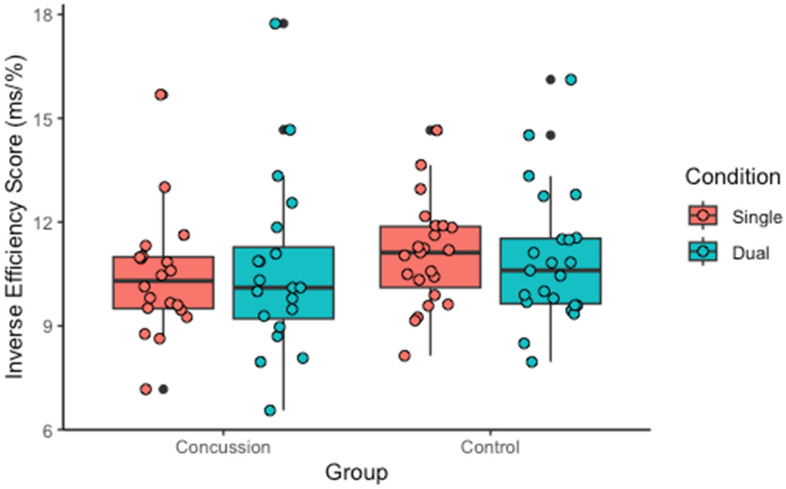
Inverse efficiency score during the single-task and dual-task conditions for the concussion and control groups.

No group x condition interaction was found for IES (*p* = 0.634). Post hoc analyses showed no effect of condition within the concussion (*p* = 0.364) and control groups (*p* = 0.378) or between-group differences for both conditions (single task: *p* = 0.110; dual tasks: *p* = 0.317), as shown in Figure 4.

No significant difference was found in the cognitive dual-task cost between the concussion and control groups (*p* = 0.634). The relative impact of the dual tasks on cognition was 2.62% for the concussion group and −0.59% for the control group. A significant correlation was found within the concussion group between time to symptom recovery and cognitive dual-task cost (r = 0.461, *p* = 0.047). No other significant association was found within the concussion group between injury-related characteristics and cognitive dual-task cost (ps ≥ 0.278).

## 4. Discussion

The present study investigated the gait and cognitive performance during challenging dual tasks of college and university athletes with post-acute concussion and in peers without a history of concussion. The results suggest no significant effect of concussion on gait speed or cognition under the dual-task condition. An important finding is the strong correlation between the gait dual-task cost and time since injury within the concussion group and the correlation between cognitive dual-task cost and time to symptom recovery.

The results indicate that the concussion group exhibited non-significant lower performance in both gait and cognitive tasks when performed simultaneously compared to individually. However, the control group demonstrated better performance (not significant and small) during the dual tasks regarding cognitive outcomes. The dual-task condition had a relative impact of 1.02% across groups, representing reduced accuracy, increased reaction time, or a combination compared to the single-task performance. Similarly, under the dual-task condition, both groups reduced their gait speed compared to under the single-task condition, with a mean relative impact of 1.34%. However, when examining the results within the concussion group only, the results showed that athletes who had more recently sustained a concussion displayed distinct behaviors under the gait-dual task condition compared to those with older concussions. Specifically, less time since injury was associated with increased gait speed, contrasting older concussions and those in the control groups, who typically reduced their speed under the dual-task condition. This suggests that the recency of a concussion may influence an athlete’s ability to manage gait under increased cognitive load, potentially reflecting varying stages of recovery.

Our findings align with several studies that did not identify a slower dual-task gait speed among concussed athletes, contrary to our hypothesis [22,23]. For example, a previous study did not find group differences between recently concussed athletes and controls on a single-task or gait-and-reading dual tasks; however, in the early stages after concussion, athletes accelerated gait speed under the dual-task condition [23]. Similarly, in our study, participants tested between 3 to 5 months after concussion showed an acceleration in gait speed under the dual-task condition, while those assessed later exhibited gait performance that had normalized to the level seen in the control group.

The fact that a relatively recent concussion selectively impacts dual-cost gait while sparing performance on the cognitive task is significant. This finding could be attributed to the allocation of attentional resources during dual-task performance as well as intrinsic motivation factors. It has been suggested that during dual-task execution, greater attentional resources are attributed to the more challenging task [18]. Additionally, the learning progress motivation hypothesis suggests that challenging tasks result in greater engagement and improved performance compared with easy tasks [24]. Hence, it is plausible that in our study, recently concussed athletes perceived the cognitive component of the dual-task as more challenging than the motor component, whereas athletes with an older concussion and controls did not perceive a difference of the same magnitude. Therefore, recently concussed athletes might have prioritized the cognitive task more, dedicating more effort and attention to it at the expense of the gait task. This prioritization could explain the consistent findings across multiple concussion studies, which report a decrease in gait dual-task speed but not in cognitive dual-task cost [25,26].

In the present study, the cognitive dual-task cost was consistent across the concussion and control groups. This finding aligns with some of the literature, which does not report differences in the cognitive dual-task cost between these groups [7,14,23]. However, many of the available studies utilized less-demanding cognitive tasks. In contrast, our research employed a switch task designed to engage higher-level executive functions, such as inhibition, flexibility, and working memory [20]. This task is sensitive to both recent [3] and older concussions in athletes who no longer report symptoms [10]. Other studies employing higher-level cognitive tasks reported increased cognitive dual-task costs in adolescents [11] and older adults with persisting symptoms after concussion [27]. Our study contributes to this body of research by including asymptomatic adult athletes, the majority of whom had recovered from their concussion within the typical recovery timeline. The lack of observed differences in dual-task performance also suggests that these athletes had achieved cognitive recovery by the time of testing.

A moderately strong significant correlation was observed between the IES dual-task cost and time to symptom recovery, indicating a potential association between the impact on cognition and concussion severity. This finding underscores the utility of these cognitive measures in identifying athletes who are at higher risk of prolonged deficits, aligning with previous studies suggesting cognitive alterations as an indicator of concussion severity [26,28,29].

Our results support the notion, as suggested by other authors [11,30], that evaluating cognitive parameters alone may not be sufficient to detect persisting deficits in athletes with concussion beyond the acute phase. Incorporating dual-task paradigms is crucial to ensure a comprehensive concussion assessment. Without this approach, some gait deficits might remain undetected, potentially increasing the risk of injury upon returning to sports. Previous research indicates that athletes who return to sports after a concussion are up to twice as likely to sustain a musculoskeletal injury [31], although the underlying causes of this increased risk are not fully understood. The possible mechanisms include alterations in gait and movement patterns, as well as neuromuscular coordination impairments, alterations of cognitive functioning, proprioceptive dysfunction, decreased physical conditioning, and lower confidence in abilities or fear of re-injury, resulting in movement dynamics alterations. While the current study did not find differences between the concussion and control groups in gait speed during the single and dual tasks, there may have been gait differences, such as changes in the center of mass movements, stride length, gait variability, step width, that were not measured herein. Future studies could investigate these gait characteristics to better understand the potential changes in gait during a dual-task challenge following a concussion.

When interpreting the present results, it is important to consider the limitations of the present study. First, the possibility of a practice effect due to repeated cognitive testing could have impacted our results. However, this was minimized by employing different yet equivalent versions of the task and by counterbalancing the order of testing [19,20]. Second, participants’ preferred walking speed may have variably affected the difficulty of the gait tasks, particularly for those who typically walk at slower speeds. However, this limitation is somewhat mitigated by the fact that preferred walking speed is correlated with height and that no statistical differences were found between these variables in our study. Lastly, the generalizability of the present results is limited to the post-acute phase of recovery and asymptomatic athletes. Outcomes may vary significantly for athletes in the acute phase or those with persisting symptoms after concussion. Prospective studies are necessary to explore the range of recovery timelines and trajectories and their respective impacts on dual-task performance.

## 5. Conclusions

Our findings highlight the added value of including dual-task conditions in assessing recovery following concussion. Increases in gait and cognition dual-task cost could indicate that athletes are not ready for a safe return to play. Future studies should investigate how this affects athletic performance, the potential for increased re-injury risks, and how acutely concussed athletes perform using our experimental dual-task protocol. Clinicians and researchers should recognize the value of integrating complex cognitive tasks in dual-task assessments. Relying on less-sensitive measures could elevate the risk of adverse outcomes for injured athletes.

## Figures and Tables

**Table 1:** Participants’ demographic information and injury characteristics.

Measures	Concussion (n = 20)	Control (n = 23)
Female sex, n (%) Male sex, n (%)	5 (25.0%) 15 (75%)	9 (39.1%) 14 (60.9%)
Age in years, mean ± SD	21.30 ± 2.41	19.91 ± 1.90
Current level of education, n (%)		
College (18–19 years)	6 (30.0%)	15 (65.2%)
University (20–25 years)	14 (70.0%)	8 (34.8%)
Years of education, mean ± SD	15.3 ± 2.66	13.8 ± 1.95
Height (cm), mean ± SD	177 ± 0.06	176 ± 0.10
Sports		
Rugby, n (%)	11 (55.0%)	5 (21.7%)
Hockey, n (%)	2 (10.0%)	0 (0.0%)
Football, n (%)	2 (10.0%)	3 (13.0%)
Soccer, n (%)	1 (5.0%)	2 (8.7%)
Volleyball, n (%)	1 (5.0%)	6 (26.1%)
Natation, n (%)	1 (5.0%)	1 (4.3%)
Basketball, n (%)	1 (5.0%)	4 (17.4%)
Cheerleading, n (%)	1 (5.0%)	2 (8.7%)
Depression symptoms (BDI), mean ± SD	5.00 ± 4.68	5.35 ± 4.58
Anxiety symptoms (BAI), mean ± SD	3.40 ± 3.50	4.48 ± 3.93
ADHD symptoms (WURS), mean ± SD	16.30 ± 12.63	12.26 ± 9.11
Injury characteristics		
Months since injury, mean ± SD	5.25 ± 0.32	-
Symptom intensity at time of injury, mean ± SD	32.65 ± 4.65	-
Number of different symptoms at time of injury, Mean ± SD	12.00 ± 0.98	-
Days to symptoms recovery, mean ± SD	21.12 ± 8.66 ^a^	-
Diagnosed concussions, ^b^ mean ± SD	2.05 ± 0.21	-
Suspected concussions, mean ± SD	0.55 ± 0.69	-

*Notes*: ADHD = attention deficit/hyperactive disorder; BAI = Beck Anxiety Inventory; BDI = Beck Depression Inventory; WURS = Wender–Utah Rating Scale. Symptom intensity and number of different symptoms were self-reported retrospectively using the Post-Concussion Symptom Scale instrument of the SCAT-5. ^a^ Except for two athletes who took 4 months to recover, athletes with a concussion achieved a full recovery within the first 3 weeks after injury; ^b^ total number of concussions, including the most recent one for which they were included in this study.

## Data Availability

The data presented in this study are available on request from the corresponding author due to ethical reasons.
